# Increased activation of the pregenual anterior cingulate cortex to citalopram challenge in migraine: an fMRI study

**DOI:** 10.1186/s12883-019-1478-0

**Published:** 2019-10-15

**Authors:** Andrea Edit Edes, Shane McKie, Edina Szabo, Gyongyi Kokonyei, Dorottya Pap, Terezia Zsombok, Mate Magyar, Eva Csepany, Gabor Hullam, Adam Gyorgy Szabo, Lajos Rudolf Kozak, Gyorgy Bagdy, Gabriella Juhasz

**Affiliations:** 1 SE-NAP2 Genetic Brain Imaging Migraine Research Group, Semmelweis University, Budapest, Hungary; 2 Department of Pharmacodynamics, Faculty of Pharmacy, Semmelweis University, Budapest, Hungary; 3 Faculty of Biological, Medical and Human Sciences Platform Sciences, Enabling Technologies and Infrastructure, Faculty of Biological, Medical and Human Sciences Research and Innovation, The University of Manchester and Manchester Academic Health Sciences Centre, Manchester, UK; 4 Doctoral School of Psychology, ELTE Eötvös Loránd University, Budapest, Hungary; 5 Institute of Psychology, ELTE Eötvös Loránd University, Budapest, Hungary; 6 Department of Neurology, Faculty of Medicine, Semmelweis University, Budapest, Hungary; 7 Department of Measurement and Information Systems, Budapest University of Technology and Economics, Faculty of Electrical Engineering and Informatics, Budapest, Hungary; 8 MR Research Center, Semmelweis University, Budapest, Hungary; 9 MTA-SE Neuropsychopharmacology and Neurochemistry Research Group, Hungarian Academy of Sciences, Semmelweis University, Budapest, Hungary; 10 Division of Neuroscience and Experimental Psychology, School of Biological Sciences, Faculty of Biological, Medical and Human Sciences, The University of Manchester and Manchester Academic Health Sciences Centre, Manchester, UK

**Keywords:** Pharmacological challenge MRI, Citalopram, Anterior cingulate cortex, Migraine without aura

## Abstract

**Background:**

The anterior cingulate cortex (ACC) is a key structure of the pain processing network. Several structural and functional alterations of this brain area have been found in migraine. In addition, altered serotonergic neurotransmission has been repeatedly implicated in the pathophysiology of migraine, although the exact mechanism is not known. Thus, our aim was to investigate the relationship between acute increase of brain serotonin (5-HT) level and the activation changes of the ACC using pharmacological challenge MRI (phMRI) in migraine patients and healthy controls.

**Methods:**

Twenty-seven pain-free healthy controls and six migraine without aura patients participated in the study. All participant attended to two phMRI sessions during which intravenous citalopram, a selective serotonin reuptake inhibitor (SSRI), or placebo (normal saline) was administered. We used region of interest analysis of ACC to compere the citalopram evoked activation changes of this area between patients and healthy participants.

**Results:**

Significant difference in ACC activation was found between control and patient groups in the right pregenual ACC (pgACC) during and after citalopram infusion compared to placebo. The extracted time-series showed that pgACC activation increased in migraine patients compared to controls, especially in the first 8–10 min of citalopram infusion.

**Conclusions:**

Our results demonstrate that a small increase in 5-HT levels can lead to increased phMRI signal in the pregenual part of the ACC that is involved in processing emotional aspects of pain. This increased sensitivity of the pgACC to increased 5-HT in migraine may contribute to recurring headache attacks and increased stress-sensitivity in migraine.

## Article highlights or key findings

- Current citalopram challenge pharmacological MRI study confirmed that migraine patients are more sensitive to the acute elevation of synaptic serotonin level.
- Citalopram challenge (e.g. acutely increased brain serotonin level) led to increased fMRI signal in the pregenual anterior cingulate cortex in migraine patients compared to controls, which is an important area processing emotional aspects of pain.
- As serotonin level shows temporary increase during migraine attacks, our results suggest that increased pregenual anterior cingulate cortex activation may contribute to the suffering element of migraine pain.

## Background

The anterior cingulate cortex (ACC) is a key structure of the pain processing network [1] as it is involved in descending pain modulation, attention to pain [2], emotional dimensions of pain and has also been implicated in the pathophysiology of pain related disorders [3]. Important structural and functional alterations have been found in this brain area in case of experimental headache, medication-overuse headache, tension-type headache and migraine [4]. A recent meta-analysis of grey matter changes in migraine showed a grey matter volume decrease in the ACC associated with headache frequency [5]. However, the specificity of grey matter changes in this region is questionable as this phenomenon has been observed in several other psychiatric and neurologic conditions [6]. Previous whole brain functional MRI (fMRI) analysis showed increased brain activation during noxious trigeminal heat stimulation specifically in the ACC in migraine patients compared to healthy controls [7]. However, another study with similar method did not find any differences between the two groups [8].

Animal studies have demonstrated that activation of excitatory synapsis in the ACC facilitate pain perception and increase the sensitivity to unpleasantness of pain [9], specifically, glutamatergic projections from the ACC enhance spinal sensory transmission which have been shown to amplify pain [10]. This excitatory neurotransmission in the ACC is modulated by serotonin, namely serotonin (5-HT, 5-hydroxytryptamine) inhibits the release of glutamate [11]. In line with these animal studies, subchronic administration of escitalopram, a selective serotonin reuptake inhibitor (SSRI), in humans led to decreased activation of the ventral ACC during anticipation of aversive vs. pleasant images in healthy subjects [12]. In addition, 7 days of fluvoxamine treatment in healthy individuals led to decreased regional cerebral blood flow in the ACC during painful stimuli [13]. These observations show the impact of serotonergic neurotransmission on the ACC functions and on corresponding pain modulation.

5-HT has been implicated in migraine pathophysiology for a long time. Several studies have found increased urinary 5-HT metabolite 5-hydoxyindoleacetic acid (5-HIAA) levels in migraine patients ictally [14, 15]. One study showed increased levels of 5-HIAA in cerebrospinal fluid in migraineurs during attacks [16]. In addition, interictally decreased plasma 5-HT levels were also observed in migraine patients [17–21]. These early observations of the altered serotonergic neurotransmission were the basis of the idea that migraine is characterized with chronically low 5-HT levels and with a temporary increase during headache. However, PET studies previously reported increased 5-HT brain levels indexed by 5-HT4 receptor binding in episodic [22, 23] and chronic [23] migraine patients, therefore the theory of low 5-HT levels in migraine has been questioned.

Serotonergic challenge is a method widely used to investigate the effect of acute increases in 5-HT level or serotonergic receptor activation due to administration of a serotonergic agent [24, 25]. Reserpine, fenfluramine and m-chlorophenylpiperazine (mCPP) are widely used to investigate serotonergic neurotransmission, however, these drugs often provoke headache in migraine patients [24]. SSRIs are able to increase brain 5-HT levels, have little affinity to other receptors [26] and rarely provoke headache in migraine [21]. Citalopram is one of the most selective SSRIs [27] and it is the only SSRI available in intravenous form, which is favored to avoid pharmacokinetic issues during the challenge. For this reason, we chose citalopram drug challenge to investigate serotonergic neurotransmission in migraine patients interictally.

The aim of our study was to investigate the acute effect of increased synaptic serotonergic levels evoked by citalopram on the ACC in migraine patients. We used subtherapeutic dose of citalopram for a drug challenge with fMRI (citalopram challenge pharmacoMRI, citalopram phMRI) to study the citalopram induced changes in the ACC activation in migraine patients and healthy subjects. We hypothesized that migraine patients would be more sensitive to the acute elevation of cortical 5-HT levels and this altered sensitivity will be shown by activation changes of the above mentioned important pain control area, the ACC.

## Methods

### Participants

Twenty-seven pain-free healthy participants (15 women, mean [SD] age = 25.8 [4.33] years, mean [SD] BMI = 22 [3]) and six migraine without aura patients (5 women, mean [SD] age = 24.3 [4.42] years, mean [SD] BMI = 20 [3]) between 18 and 50 years of age were recruited through advertisement.

Trained researchers screened all participants for their eligibility. The participants then underwent a medical examination by the neurologist and psychiatrist researchers.

Exclusion criteria were current or past serious medical, major psychiatric or neurologic disorders and left-handedness, use of daily medication except contraceptives, and in case of migraine patients the use of preventive medications. Mini-International Neuropsychiatric Interview (M.I.N.I.) was used to exclude any mental disorders [28]. Volunteers who had any history of excessive alcohol consumption or psychotropic medication use were also excluded. All participants were asked to refrain from caffeine intake for 4 h and from alcohol intake for 24 h prior to the MRI sessions. All participants were pain- and medication-free in the preceding 48 h before each scanning session.

The diagnosis of migraine without aura was made by neurologist researchers according to the International Headache Society criteria [29]. The study was approved by the Scientific and Research Ethics Committee of the Medical Research Council, Budapest, Hungary. After receiving written and oral information all subjects gave written informed consent. The study was carried out in accordance with the principles of Declaration of Helsinki.

### Headache characteristics of the participants

Participants with migraine without aura: The mean duration of the disorder [SD] was 11 years [4.56] with mean number of migraine attacks per month [SD] = 4.75 [2.99]. Three of six patients used sumatriptan. All migraine patients used analgesics including metamizole, ibuprofen, paracetamol, diclofenac, or paracetamol –propyphenazone – caffeine containing combined tablet for treating their headaches, and 2 of them also used domperidon to prevent nausea and vomiting. All of the migraine patients reported occasional tension type headaches. None of the migraine subjects suffered from medication-overuse headache and/or chronic migraine. The mean [SD] time of the preceding migraine attack before the placebo scans was 6 [3.6] days, and before the citalopram scans was 9 [4.7] days. All migraine patients were headache- and medication-free in the preceding 48 h before each scanning session.

#### Healthy controls

All healthy participants reported less than 12 mild tension type headache per year with no migraine type symptoms.

### Experimental design

All subjects attended two 30 min-lasting phMRI scanning sessions, separated by at least 2 weeks. Anatomical datasets were acquired during the first occasion. During phMRI sessions all subjects received placebo (normal saline) or 7.5 mg citalopram infusion in a double-blind, randomized, balanced order design. All subjects were cannulated at least 45 min before they entered the scanner. A 10 min saline infusion was followed by a saline or citalopram infusion which lasted for 7.5 min. In the remaining time a saline infusion was used to keep vein open. In the scanner all participants rested viewing a blank screen and they received yes/no questions every 5 min to monitor their current state. Participants could respond with button press (1 = yes, 2 = no) for the statements: anxious, drowsy, lightheaded, nauseous, restless and uncomfortable.

### Data acquisition

Functional dataset acquisition was performed on a 3 Tesla MRI scanner (Achieva 3 T, Philips Medical System) using a T2*-weighted echo-planar (EPI) pulse-sequence (TR = 2.500 ms, TE = 30 ms, FOV: 240 × 240 mm2 and with 3x3x3 mm resolution) using an eight-channel SENSE head coil. Anatomical images were acquired using a 3D T1-weighted turbo field echo (TFE) sequence and 1x1x1 mm resolution.

### Subjective state data analysis

The subjective states in the healthy and patient groups were analysed in SPSS 25 (IBM Corp. SPSS Statistics for Windows, Version 25.0) using Wilcoxon signed-rank tests and Mann-Whitney tests to determine any differences between the control and patient groups at *p* < 0.05 significance level.

### fMRI data analysis

Imaging data analysis was performed using Statistical Parametric Mapping (SPM 12, Friston, The Welcome Department of Cognitive Neurology, London, UK). All images were realigned using the first image as reference, then these images were spatially normalised into stereotactic MNI space and then smoothed with a Gaussian kernel of 8 mm FWHM.

ARtifact detection Tools (ART) toolbox (http://www.nitrc.org/projects/artifact_detect/) for SPM was used to determine movement artefacts in the scanner. We defined outliers as time points in which global signal deviated more than 3 SDs from the mean or motion to any direction that exceeded 1 mm deviation. Exclusion criteria was more than 15% of outliers in the whole session. All patients and healthy controls satisfied these criteria.

During first level analysis the phMRI scans were divided into 30 consecutive 1 min time-bins (T01 to T30; the 1 min time bins were used to investigate the activation changes over time due to the drug challenge). The images were then normalised by subtraction of the T01 time bin from the other 29 time bins (T02-T30) and the images were then controlled for signal drift using a nonlinear model of the drift. The time-bin images for the saline scans were then subtracted from the corresponding time-bins for the citalopram scans. The baseline time-bin (T10, as it is not biased by the signal variations in the first 10 min before citalopram administration) and T11-T30 post-infusion time bins were then entered into a 2 by 21 repeated measures ANOVA with time as a repeated measure factor and diagnosis as a grouping factor. We investigated the time by treatment by group interaction in the ACC, defined according to the Talairach Daemon (TD) atlas label that contains both left and right ACC. Results are reported at a small volume corrected peak level threshold of Family Wise Error corrected p(FWE) < 0.05.

## Results

### Behavioral and migraine related data

The number of ‘yes’ answers to questions about current subjective state did not differ significantly between the saline and citalopram challenge according to the Wilcoxon test in 27 healthy subjects and in 6 migraine without aura patients (Table 1.).

**Table 1:** The results of Wilcoxon signed-rank test of ‘yes’ answers to questions about subjective states between citalopram and placebo sessions in control subjects (CO) and migraine patients (M)

	Anxious		Drowsy		Lightheaded		Nauseous		Restless		Uncomfortable	
	Z	p	Z	p	Z	p	Z	p	Z	p	Z	p
CO	−1.069	0.285	−1.212	0.226	−1.826	0.068	−1.633	0.102	−0.197	0.844	−0.104	0.917
M	0.000	1.000	−1.265	0.206	−1.342	0.180	−1.342	0.180	−1.272	0.785	0.000	1.000

Mann-Whitney tests showed no significant difference between the two groups in neither subjective answers during citalopram phMRI sessions (Table 2.).

**Table 2:** Results of Mann-Whitney tests comparing healthy and migraine groups based on answers about subjective states during citalopram session

Grouping variable	Anxious		Drowsy		Lightheaded		Nauseous		Restless		Uncomfortable	
	Z	p	Z	p	Z	p	Z	p	Z	p	Z	p
Diagnoses	−0.471	0.637	−0.688	0.492	−1.214	0.225	−1.347	0.178	−0.258	0.796	−1.553	0.121

No migraine patients experienced headache during the scanning sessions. No subject reported headache after citalopram session in the following 72 h. Two subjects reported migraine attack 24 and 48 h after placebo session.

### Citalopram challenge phMRI

We found significant difference in ACC activation between control and patient groups in two peaks in the right pregenual ACC (pgACC) during and after citalopram infusion compared with placebo. In addition, a trend of significant difference between groups was found in the left pregenual part of rostral ACC (Table 3.). The extracted time-series showed that the activation of ACC increased in migraine patients compared to controls, especially in the first 8–10 min after the beginning of the citalopram infusion.

**Table 3:** ROI analysis results of control versus patient groups comparison in the citalopram minus placebo data at *p* < 0.001 uncorrected height threshold. (^*^significant clusters after Family Wise Error correction at p(FWE) < 0.05 secondary threshold; k = number of voxels)

MNI coordinates			side	k	p(FWE)	*F* value
x	y	z				
15	38	8	right	9	0.006	2.969^*^
9	41	−4	right	6	0.022	2.745^*^
−9	44	8	left	6	0.075	2.519

**Fig. 1:**
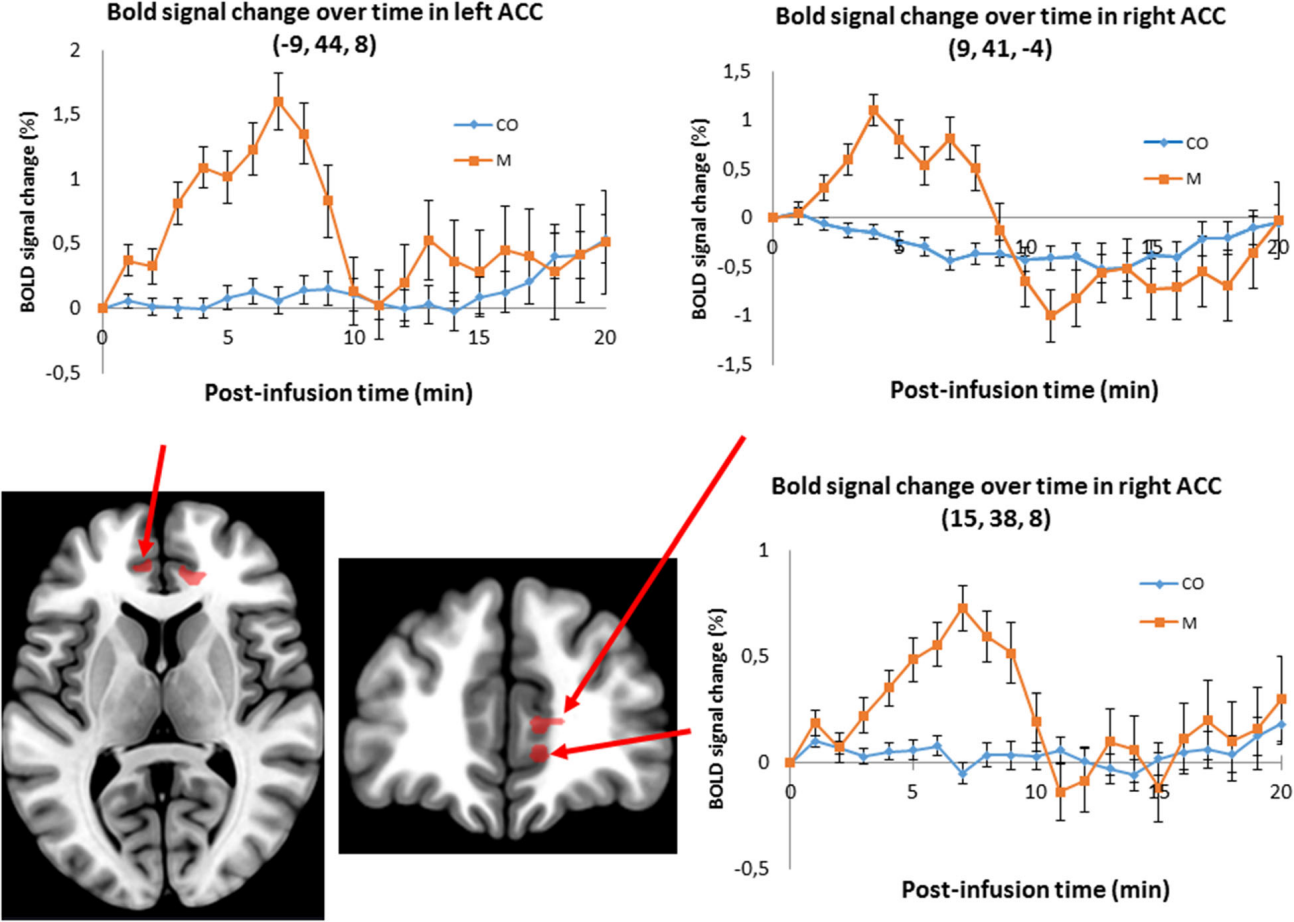
BOLD signal changes over time after citalopram minus placebo data extraction in three clusters of the ACC with error bars (standard error of mean), for control and patient groups, respectively. Coronal and sagittal view of ROI analysis results were made by p(FWE) < 0.05 secondary threshold (with *p* < 0.001 primary threshold, k ≥ 5, CO = control group, M = migraine group)

Blood oxygen level dependent (BOLD) signal changes over time are illustrated for the three pgACC cluster peaks in Fig. 1.

## Discussion

In this pilot study, using citalopram phMRI we demonstrated a significant difference in temporal activation pattern of the ACC between healthy control participants and migraine without aura patients during the acutely increased extracellular 5-HT level in the brain. The intravenous citalopram elicited increased activation over time in two peaks within the pregenual part of the right ACC in migraine patients when compared to healthy controls. This increased activation in migraine subjects was more pronounced in the first 10 min after the start of the citalopram. In addition, increased activation was found in one cluster in the left rostral ACC in migraine patients. Though this latter activation did not survive the correction for multiple comparisons, our results suggests a bilateral effect of increased brain 5-HT level on the ACC in the migraine patients.

The altered functions of the pgACC in migraine have been reported in previous fMRI studies. Increased activation was found in migraine patients to trigemino-nociceptive stimulation compared to controls also in the rostral part of the ACC [30]. Emphasizing the importance of our results, previous fMRI studies using trigeminal heat stimulation to induce acute pain in migraine patients, found increased brain activation in the pgACC with almost identical MNI coordinates as reported here [8, 31]. One of these studies showed that this enhanced activation to noxious heat reduced after a 60 day successful treatment with external trigeminal neurostimulation [31]. The authors suggested that the pgACC could be involved in the antinociceptive effect of the anti-migraine treatment [31]. Our results are in line with these results as we found that this region is more active in migraine patients after a small increase of 5-HT levels compared to pain-free healthy controls. These observations together suggest that increased sensitivity of the ACC in migraine patients might be related to altered serotonergic control of the incoming excitatory signals.

According to literature the most consistently reported area of cingulate cortex to painful stimuli is the dorsal ACC or anterior middle cingulate cortex (aMCC). The activation of this area is one of the earliest responses to pain as this region has an impact in cortical nociception [32]. A recent meta-analysis showed a significant likelihood of activation to painful stimuli in this brain area in healthy subjects [33]. In case of our study we did not find any activation of this area, however, we have not used any painful stimuli during the scanning sessions and we investigated the difference between migraine patients and healthy controls.

On the basis of the functional imaging studies of the last 20 years, the role of pgACC in many aspects of emotional processing seems to be established. In a PET study the pgACC was active during the assessment of internal emotional state induced by emotional pictures with different valences [34]. Another study with fMRI showed that the subjective ratings of pleasantness or aversiveness of sensory stimuli correlated with the activation of this area during a decision making task [35]. In addition, the pregenual part of ACC is a central node of the default mode network, a task-negative network that is consistently active during mind-wandering and it is implicated in the affective network [36]. The pgACC is the only area in the cingulate gyrus that has connection with all other regions of the cingulate cortex therefore this ACC subregion could be considered as the anterior cingulate association area [36], which is involved – along with other brain areas – in integrating information across the brain [37]. These observations point to the essential role of the pgACC in emotional processing, specifically in emotional awareness, e.g. the fundamental role of this area for individuals to assess their own emotional experience [38]. Thus, our results may suggest that migraine patients have exaggerated responses to interoceptive, e.g. emotional or visceroceptive stimuli that might be related to altered serotonergic neurotransmission in migraine. However, taking into account the rich brain network of the pgACC further studies are needed to determine whether the increased 5-HT level in the brain directly sensitizes the pgACC in migraineurs or the increased activation of the ACC is secondary to a complex interplay between the increased 5-HT level and those subcortical and other cortical areas that are important in processing different sensory and interoceptive signals.

Nevertheless, the pgACC is the ACC subregion with the highest opioid receptor density [39] and it also plays an important role in opioid analgesia and opioid placebo effect [40]. In addition, the increased pgACC signal during noxious stimuli may reflect the attention to unpleasantness of pain [41, 42]. The recurring headache attacks and the increased attention to unpleasantness of pain in migraine patients may cause altered pgACC functions. Our results extend this observation and suggest that this phenomenon might be related to altered serotonergic neurotransmission in migraine.

A recent systematic review of electrophysiological and neuroimaging studies of serotonergic system in migraine confirmed the altered 5-HT neurotransmission which has been a main area of interest in migraine research for decades [43]. The authors supported the notion of suddenly increasing 5-HT levels during migraine headache [43]. Based on our results we speculate that during migraine attack the pgACC reacts to the spontaneously elevated 5-HT levels the same way as in our study by citalopram challenge i.e. with increased activation. If so, it may contribute to the development of migraine attack by facilitating the pain transmission at the trigeminal level [44] similarly to that observed at the spinal cord [10].

In line with previous studies, we can conclude that migraine patients are more sensitive to acute increase in 5-HT levels and that this phenomenon can be observed in a subregion of the ACC which is involved in emotional aspects and suffering elements of pain and may be involved in the modulation and/or chronification of migraine. However, the direction of the connection between migraine attacks and the steep increase of 5-HT levels during headache remains unclear.

Interestingly, a previous study of peripherial neurochemical changes in migraine showed that slight release of platelet 5-HT after a nitroglycerin test could be protective against migraine development [19]. The authors reported that no migraine attack developed in migraine patients who responded with increased peripherial 5-HT level to nitroglycerin [19]. However, this study investigated only the peripherial changes of 5-HT level. In addition, nitroglycerine induced migraine may develop through other mechanisms than migraine attacks caused by 5-HT releasing agents. As 5-HT releasing agents e.g. reserpine can provoke migraine [24], it is possible that migraine attacks caused by extensive increase in brain 5-HT levels in sensitive patients may be related to the increased activation and altered pain-modulation of the pgACC.

Nevertheless, it has to be mentioned that none of our 6 migraine patients developed headache during or immediately after the citalopram administration. As we used a relatively low dose of citalopram the increase in 5-HT levels probably did not reach the level that occurs during migraine attack.

Our major limitation in this study is the relatively low number of participants in the patient group. Despite the low sample size, we found significant activation difference between the two groups. It would be fruitful however to investigate the acute effect of 5-HT level changes on the ACC activation in more migraine patients in the future to replicate and confirm our results. It would be also important to investigate the activation differences between migraine patients and controls in other important pain processing areas or even in the whole brain with higher sample size.

Furthermore, it has to be mentioned that in contrast to PET phMRI is not suitable to detect specific receptor activation changes, therefore we were not able to determine which serotonergic receptor or receptors are responsible for the observed activation changes. However, our aim was not to detect changes in receptor activation, rather to investigate the general sensitivity to increased 5-HT levels in migraine.

In addition, we have not corrected our analysis for potential individual differences of the grey matter density in the ACC that might influence the BOLD signal changes.

Finally, in spite of the fact that the pgACC is highly involved in emotional processing, we did not find any difference between citalopram and placebo sessions and between migraine and control groups in the subjective states. Thus, the difference between the two groups to citalopram could not be detected at subjective behavioral level, only at neural level. However, the lack of changes at behavioral level could be related to the dichotomous characteristic of the answers or to the relatively low dosage of citalopram.

## Conclusions

In conclusion, our findings confirmed the increased sensitivity to elevated 5-HT and the altered functions of the ACC in interictal migraine patients compared to controls. Our results demonstrate that a small increase in 5-HT level leads to increased activation in the pregenual part of the ACC which has been previously reported to be involved in emotional aspects of pain. These observations suggest that the pgACC activation might also increases during migraine attacks, which process might be related to the suddenly increasing 5-HT levels, contributing to the suffering element of pain. The increased sensitivity of the pgACC in migraine patients may also contribute to the recurring migraine attacks and the increased stress-sensitivity in migraine.

Finally, our study offers a new approach to investigate the brain of migraine patients: we proved the acute citalopram challenge is a suitable method to detect functional alterations in migraine. This method could deepen our understanding about the pathophysiology of migraine and even other neurologic or mental disorders in the future that are related to altered 5-HT neurotransmission.

## Data Availability

Data are available upon request for researchers.
